# Single center experience with carotico-subclavian bypass

**DOI:** 10.1186/1749-8090-8-S1-O112

**Published:** 2013-09-11

**Authors:** A Kucuker, M Hidiroglu, L Cetin, A Kunt, F Saglam, M Canyigit, E Sener

**Affiliations:** 1 Cardiovascular Surgery Department, Izmir Katip Celebi University, Ataturk Training and Research Hospital, Karsiyaka-Izmir, Turkey; 2 Radiology Department, Izmir Katip Celebi University, Ataturk Training and Research Hospital, Karsiyaka-Izmir, Turkey

## Background

We present our patients we treated with carotico-subclavian bypass operation for subclavian steal syndrome or as an adjunctive procedure to thoracic endovascular stent- grafting (TEVAR) procedures necessiating left subclavian artery occlusion intentionally.

## Methods

We performed 11 carotico-subclavian bypass operations between August 2009-January 2013 at our department. 8 patients were operated for subclavian steal syndrome with subclavian artery occlusion and 3 patients were operated before TEVAR for aortic dissection/aneursym necessiating left subclavian artery coverage. 9 male (81.81%) and 2 female (18.18%) patient with the age between 55-73 (mean 64.9) were operated. 8mm Dacron graft was used for nine patients and 7/8mm PTFE for two patients. Anastomosis were done in end-to-side fashion.

## Results

Radial artery pulses were palpabl in all patients after the operation with resolving complaints as left arm pain or dizziness.1patient with native carotis artery tortiosity and cerebral artery aneursym suffered graft thrombosis 2 days after the operation (he could not be anticoagulated). He was reoperated and graft was cut near carotis anastomosis. After removing thrombotic material and providing flow inside graft, reanastomosis to a more distal and straight part was performed. No neurological complications were observed. 1 patient suffered puffiness and pain at the left shoulder two weeks after operation because of seroma that was drained percutaneously with ultrasonography guidance. Patients to have TEVAR procedures were primarily operated for carotico-subclavian graft interposition and subsequently TEVAR was performed at the same stage. The mean follow-up period is 20 months with all grafts having patency on ultrasonographic examination.

## Conclusion

Carotico-subclavian bypass is a safe procedure with good surgical results.

